# Interpolymer Complexes Based on Cellulose Ethers: Application

**DOI:** 10.3390/polym15153326

**Published:** 2023-08-07

**Authors:** Raushan Keldibekova, Symbat Suleimenova, Gulden Nurgozhina, Eldar Kopishev

**Affiliations:** 1 Faculty of Natural Sciences, Department of Chemistry, L.N. Gumilyov Eurasian National University, Astana 010000, Kazakhstan; keldibekova_rn_2@enu.kz (R.K.);; 2 Faculty of Natural Sciences, Department of General and Inorganic Chemistry, Bukhara State University, Bukhara 705018, Uzbekistan

**Keywords:** interpolymer complexes, cellulose ethers, mucoadhesion, hydrogels, drug delivery

## Abstract

Interpolymer complexes based on cellulose ethers have gained significant interest in recent years due to their versatile applications. These complexes are formed by combining different polymers through non-covalent interactions, resulting in stable structures. This article provides an overview of the various fields where IPCs based on cellulose ethers find application. IPCs based on cellulose ethers show great potential in drug delivery systems. These complexes can encapsulate drugs and enable controlled release, making them suitable for sustained drug delivery. They offer advantages in terms of precise dosage and enhanced therapeutic efficacy. Coatings and adhesives also benefit from IPCs based on cellulose ethers. These complexes can form films with excellent mechanical strength and enhanced water resistance, providing durability and protection. They have applications in various industries where coatings and adhesives play a crucial role. In food packaging, IPCs based on cellulose ethers are highly relevant. These complexes can form films with effective barrier properties against oxygen and water vapor, making them ideal for packaging perishable foods. They help extend to shelf life of food products by minimizing moisture and oxygen transfer. Various methods, such as solvent casting, coacervation, and electrostatic complexation, are employed to synthesize IPCs based on cellulose ethers.

## 1. Introduction

The initial reference to interpolymer complexes (IPCs) (also referred to as polymer-polymer complexes in some sources) can be traced back to the research documented in [1]. Subsequently, Bungenberg de Jong extended these investigations, as indicated in references [2,3,4,5]. The systematic studies in this field were first undertaken by Liquori, as evidenced by works [6,7]. During the period of 1970–1980, extensive research was conducted, which established the fundamental basis for the subsequent rapid progress in the study and application of interpolymer complexes. Notable contributions in this era were made by prominent scientists, such as Bekturov [8], Zezin [9], Papisov [10], Izumrudov, Kabanov [11], and Tsuchida [12], who played pivotal roles in leading these scientific endeavours. 

IPCs exhibit a complex structural arrangement, which arises from the interaction between macromolecules of polymers possessing complementary chemical structures when in a solution. The characteristics of macromolecules are determined not only by the chemical structure of the polymer chain but also by the formation of macromolecular aggregates. Thus, the macromolecules exhibit higher-order structures, including the configuration and conformation of the polymer chains, in addition to their primary structures. The effects of aggregation are typically observed as phase separation phenomena, such as precipitation, gelation, coacervation, emulsification, as well as the crystallization and liquid crystallization of polymers, or the self-assembly of biopolymer subunits. At present, IPCs are recognized as highly promising materials for their ability to act as structure-forming agents in various types of dispersions [13,14,15,16].

The investigation into the growth of interpolymer complex (IPC) films is of significant interest both from a scientific perspective and for practical applications. The cooperative interaction of complementary structures found in IPCs plays a crucial role in chemistry, polymer physics, and molecular biology. Despite the extensive research conducted on interpolymer complexes, there remain unanswered questions concerning their experimental and theoretical data, highlighting the need for further scientific advancements in this field.

A comprehensive analysis of the available data leads to the following conclusions: the primary focus of IPC research lies in understanding the material’s structural construction, the physicochemical parameters that influence polycomplex growth, and the diverse range of applications for IPCs. Essential factors influencing the structural growth of IPCs include solution pH, the temperature at which functional group binding occurs, solution concentration, the presence of low molecular weight compounds, and other relevant parameters.

**Figure 1:**
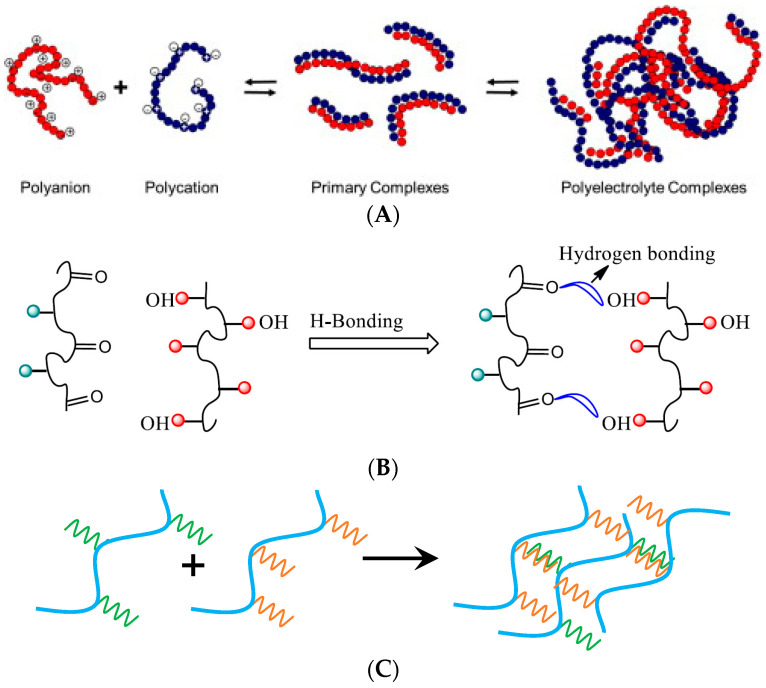

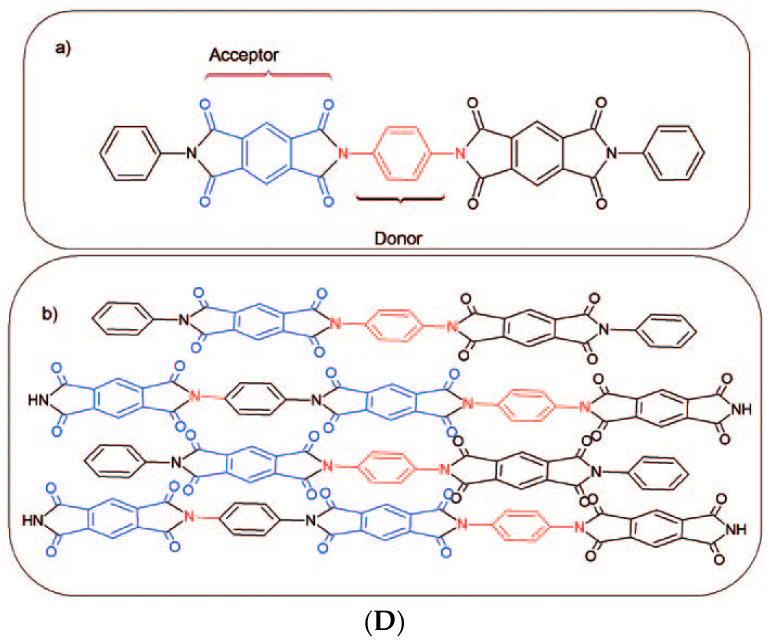
Four classes of IPCs. (**A**) Formation of polyelectrolyte complexes [20]. (**B**) Formation of hydrogen bonded complexes [25]. (**C**) Stereocomplexes (based on [25]). (**D**) Charge transfer complexes [27], intramolecular (**a**) and intermolecular (**b**) structures.

Based on the forces of interaction involved, intermacromolecular complexes can be categorized into the following four distinct classes (Figure 1):

- Polyelectrolyte complexes [17,18,19,20];
- Complexes of hydrogen bonds [12,21,22,23];
- Stereocomplexes [6,24,25];
- Charge transfer complexes [26,27].

## 2. Methods of Obtaining

Several methods have been established for obtaining interpolymer complexes, including the following:

- Mixing complementary macromolecules in solutions [28,29,30,31,32];
- Matrix polymerization [33,34,35,36,37];
- Interactions at the boundary of liquid compounds [38,39,40,41];
- Application methods on solid and flexible surfaces [42,43,44,45].

**Figure 2:**
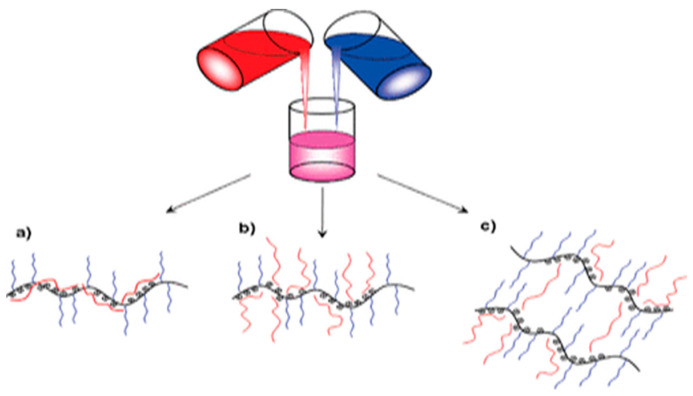
Mixing complementary macromolecules in solutions [32]. (**a**) Poly(sodium styrenesulfonate (PSS) is buried among the poly(ethylene oxide) brushes, (**b**) extended PSS chains from complex core, and (**c**) PSS chains bridging between complexes.

Among these approaches, one of the feasible and reliable methods for creating interpolymer complexes involves employing joint assembly processes within multicomponent polymer systems that contain complementary macromolecular components. Specifically, electrostatically controlled joint assembly is utilized, where water solutions of oppositely charged polyelectrolytes are mixed, leading to the formation of IPCs (Figure 2). These complexes represent macromolecular co-assemblies stabilized by an interconnected network of interpolymer salt bonds [28].

**Figure 3:**
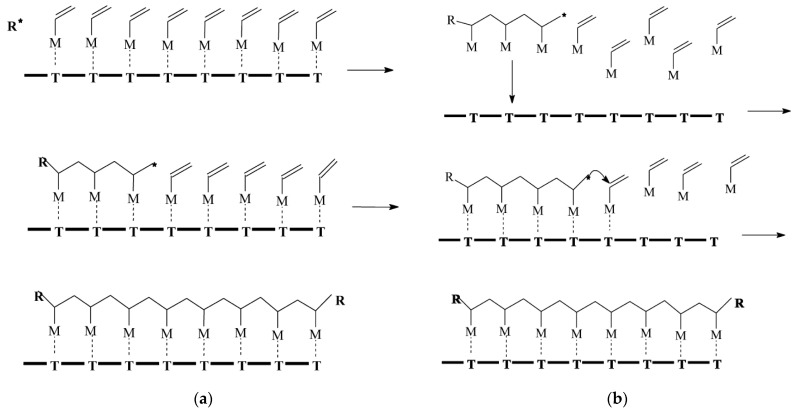
Example of matrix polymerization [34]. (**a**) The ‘zip’ mechanism, *—electrostatic, hydrogen bridges and (**b**) ‘pick-up’ mechanism, *—oligomer with critical length.

Matrix polymerization is an intriguing approach based on the conventional matrix synthesis model, which offers a means to synthesize composite materials that may be challenging or unattainable using alternative methods. Originally, the method was denoted as rreplica polymerization [46]; subsequently, it was assigned two additional designations, namely, “Template polymerization” and “Matrix polymerization”, which ultimately superseded the former term. The interpolymer complexes obtained through this method exhibit sufficient stability, and their polymer components can only be separated by disrupting the cooperative systems of intermolecular bonds [35]. Połowiński [34] differentiates between two distinct mechanisms of polymerization (Figure 3).

**Figure 4:**
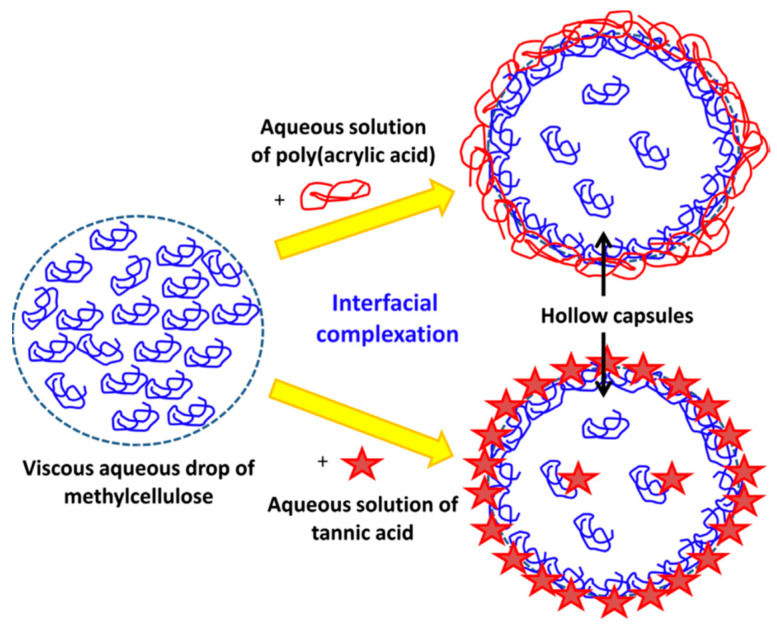
Example of interfacial complexation [41].

Another method for obtaining IPCs involves conducting a reaction at the interface of two immiscible liquid compounds. This technique relies on two solutions containing complementary polymers in separate solvents, resulting in the formation of a thin film of IPC at the phase boundary (Figure 4) [38].

**Figure 5:**
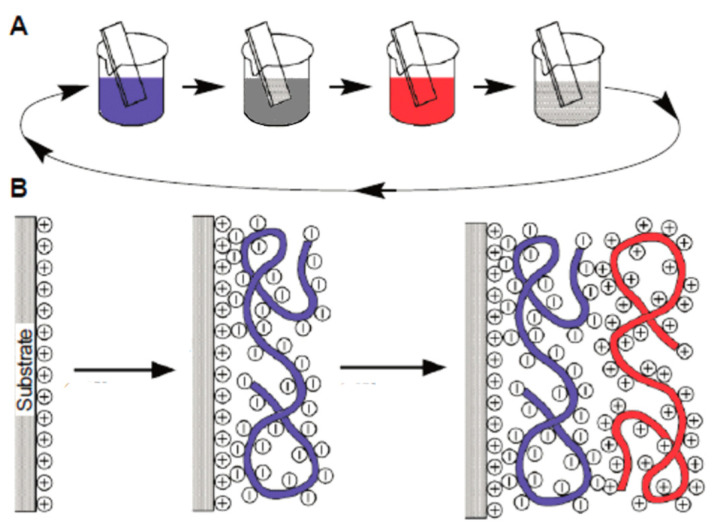
Application methods on solid and flexible surfaces [45]. (**A**) Schematic of the film deposition process using slides and beakers. (**B**) Simplified molecular picture of the first two adsorption steps, depicting film deposition starting with a positively charged substrate. The molecules and solutions of the polyanion and polycation are distinguished by the utilization of red and blue colors, respectively.

The application method on both solid and flexible surfaces entails the sequential deposition of polymer solutions capable of forming soluble and insoluble interpolymer complexes. This approach offers notable advantages, including precise control over coating thickness and the ability to develop materials with tailored physicochemical properties. Multilayer coatings can be applied to various surfaces, holding potential applications in the medical field and other scientific disciplines (Figure 5) [43,45].

## 3. Methods of Studying IPCs

Throughout this research, a substantial body of scientific literature on interpolymer complexes and their preparation and analysis methods has been thoroughly examined. Notably, standardized approaches exist for investigating the partially formed complexes, including the following:

- Titration [47];
- Viscometry and turbidimetry [48];
- Potentiometry [49];
- IR spectroscopy [50];
- Differential scanning calorimetry [50];
- Scanning electron microscopy [51];
- Dynamic light scattering [52];
- Three-dimensional (3D) integrated optics [53];
- Static light scattering [54], etc.

## 4. IPCs Based on Cellulose Ethers

This review primarily examines interpolymer complexes (IPCs) derived from cellulose ethers as its main subject of interest.

Cellulose ethers are derivatives of cellulose with a general chemical formula [C_6_H_7_O_2_(OH)_3−x_(OR)_x_]_n_, where n represents the degree of polymerization. In this formula, x denotes the number of hydroxyl (OH) groups substituted in a single unit of the cellulose macromolecule, while R represents an alkyl, acyl, or mineral acid residue. Each unit of the macromolecule contains three hydroxyl groups, which have the capability to undergo reactions to form ethers and esters. In the case of mixed cellulose esters, different substitutive radicals are present.

**Figure 6:**
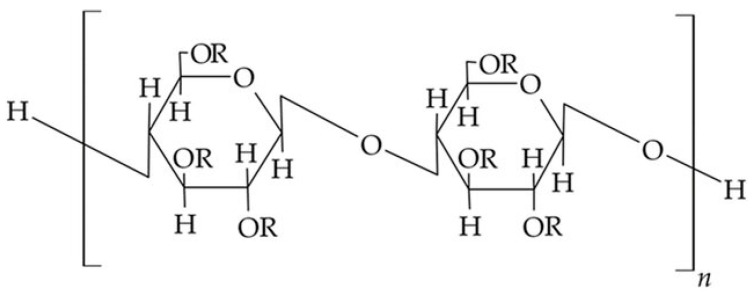
Chemical structure of cellulose esters/ether derivatives [55].

The most commonly encountered cellulose ethers and esters include ethers, such as carboxymethylcellulose, methylcellulose, ethyl cellulose, as well as methylhydroxypropyl cellulose, oxypropyl cellulose, and cyanethyl cellulose. Esters include cellulose acetates, cellulose nitrates, as well as acetylphthalyl cellulose, acetopropionates, acetobutyrates, and cellulose sulfates (Figure 6).

Cellulosic materials encompass a variety of cellulose derivatives, including cellulose esters, such as acetate, acetate trimellitate, acetate phthalate (CAP), hydroxypropyl methyl (HPM, also known as hypromellose) phthalate, and HPM acetate succinate. These esters are formed through the esterification of hydroxyl groups in cellulose using acetic, trimellitic, dicarboxylic phthalic, and succinic acids, or a combination of them.

Additionally, cellulosic materials also comprise cellulose ethers, such as methyl, ethyl, hydroxyethyl, hydroxyethyl methyl, hydroxypropyl (HP), HPM, and carboxymethyl ethers of cellulose. These ethers are formed by etherification of hydroxyl groups in cellulose using the appropriate alkyl halide (R-Cl) after the alkalization of cellulose, typically derived from wood pulp (Table 1).

The properties of cellulose ethers are primarily influenced by factors, such as the number, degree of substitution, and type of substituent R. The degree of polymerization, typically ranging from 150 to 500, significantly impacts the strength and viscosity properties of cellulose ethers, allowing for a wide range of applications. The physico-mechanical and chemical properties are determined by the degree of substitution. The average degree of substitution, denoted as γ, falls within the range of 0 to 3. However, it is often calculated for 100 elementary units of cellulose macromolecules (e.g., cellulose triacetate with γ = 280–290). The degree of substitution can be controlled by adjusting synthesis conditions, such as the concentration of alkylating or esterifying agents, temperature, and duration.

The solubility of cellulose ethers relies on the composition and ratio of substituents and free hydroxyl (OH) groups. For instance, cellulose acetate, with a degree of substitution of 0.5–0.8 and 1.5–1.8, is, respectively, soluble in water and an acetone–water mixture (7:3). Cellulose acetate with a degree of substitution of 2.2–2.6 is soluble in acetone, while methyl cellulose, with a degree of substitution exceeding 2.6, dissolves in methylene chloride and a mixture of methylene chloride-ethanol (9:1). As the alkyl radical’s chain length increases, the hydrophobicity of cellulose ethers rises, enabling solubility in nonpolar solvents (e.g., butyl- and propyl cellulose are insoluble in water but soluble in organic solvents). The solubility of cellulose ethers in organic solvents tends to increase with higher temperatures but decreases with greater molecular weight.

As the number of carbon atoms in the substituent increases, several properties of cellulose ethers undergo changes. These include a decrease in moisture absorption, as well as reductions in softening and melting temperatures. It is worth noting that esters exhibit thermal instability and possess low resistance to acids and alkalis. In contrast, ethers demonstrate stability when exposed to acids and alkalis, and they can withstand relatively high temperatures without decomposing or releasing free acids that may lead to metal corrosion. Both cellulose ethers and esters exhibit good dielectric properties.

## 5. Application of IPCs

**Figure 7:**
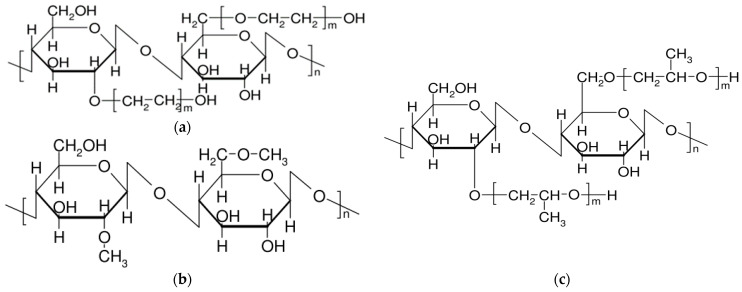
Structure of cellulose ethers. (**a**) Hydroxyethyl cellulose. (**b**) Methylcellulose. (**c**) Hydroxypropyl cellulose.

Cellulose ethers find extensive application in the manufacturing of contemporary dry building mixes (such as hydroxyethyl methyl cellulose, HEMC, and hydroxypropyl methyl cellulose, HPMC), which enhance water retention, thickening, and improve the processability of the mixture (Figure 7). They are also utilized in the production of pharmaceutical dosage forms. These complexes offer the advantage of prolonging the residence time of dosage forms in mucous membranes, leading to increased bioavailability of medications (Table 2).

Irrespective of the route of drug administration, such as nasal, oral, ocular, or rectal, mucoadhesive drug delivery systems are considered favorable and versatile in the formulation of dosage forms.

Certain polymer structural characteristics necessary for mucoadhesion can be summarized as follows: the presence of strong hydrogen bonding groups, significant anionic or cationic charges, high molecular weight, chain flexibility, and surface energy properties that facilitate spreading on mucus.

A review of the works conducted by V.V. Khutoryansky and colleagues [56,78,79,80,81] focused on the development of hydrophilic and hydrophobic films using combinations of polycarboxylic acids, polysaccharides, and various nonionic polymers. These multilayer materials composed of synthetic polymers and natural nonionic polysaccharides have gained considerable interest due to their excellent biocompatibility and potential biomedical applications. The resulting films exhibited a wide range of glass transition temperatures and mechanical properties, ranging from glassy polymers to highly elastic rubber-like materials that displayed greater susceptibility to deformation compared to other counterparts. The unique properties of these interpolymer complexes (IPCs) hold promise for the development of novel bioadhesive drug delivery systems, particularly in situations where the material must possess sufficient softness to prevent damage to living tissues, such as in ophthalmology [74]. Notably, several articles [82,83] examined the mucoadhesive potential of soluble and cross-linked multilayer hydrogel films and evaluated their biocompatibility in the mucous membrane of pig cheeks. The authors concluded that the developed films have potential applications in the local delivery of anesthetics in dental treatments.

In a related study [31], efforts were made to develop novel formulations containing metronidazole for the treatment of ulcerative wounds. Flour-adhesive tablets were prepared by employing different combinations of cellulose and polyacrylic derivatives. The tablets underwent characterization through investigations of swelling, mucoadhesion time, and drug release. Results revealed that these tablets exhibited prolonged local release of metronidazole in the oral cavity for a duration of 12 h, along with a significant reduction in the daily dosage compared to traditional systemic therapy.

Similarly, Musial [71] conducted research in 2007 on the release rate of metronidazole from hydrogels for the treatment of acne rosacea. The influence of various acrylic acid polymers in conjunction with methylcellulose on the release rate of metronidazole from hydrogels was examined. Compositions containing Carbopol 971P and methylcellulose demonstrated an increase in viscosity within a specific range of methylcellulose concentration. The drug release process in all formulations exhibited a two-stage pattern. Among the biopolymer formulations, Carbopol 980NF with methylcellulose exhibited the highest release rate during the first stage. These gels with comparable rheological properties can be utilized for ex vivo and in vivo studies to achieve optimal drug activity of metronidazole in the treatment of inflammatory skin conditions.

Furthermore, in another study [57], researchers aimed to develop buccal tablets for the delivery of chlorhexidine diacetate, an antimicrobial drug widely used for periodontal disease treatment. Mesoporous silicate (MCM-41) was incorporated into the tablets by combining an aqueous solution of sodium metasilicate with an aqueous solution of cetyltrimethylammonium chloride, following the method described in reference [84]. Mucoadhesive polymers were employed in combination with MCM-41 to prepare the mucoadhesive tablets. Different mixtures of hydroxyethyl cellulose (HEC)/MCM-41 and carboxymethylcellulose/MCM-41 were utilized. The tablets were evaluated for their swelling behavior, ex vivo mucoadhesion time, and strength. Tablets formulated with a ratio of 2:1 HEC/MCM-41 exhibited the most favorable mucoadhesive properties, efficient drug release, and effective antifungal activity, indicating the suitability of HEC as the starting polymer for the preparation of interpolymer complexes (IPCs).

In a study conducted by Majid Saidi et al. [58], the influence of polyvalent cations on the release of theophylline from tablets made of polyanionic polymers, specifically sodium alginate and sodium carboxymethylcellulose, was investigated. Theophylline is commonly used as a bronchodilator for the treatment of chronic obstructive pulmonary disease, making it crucial to identify optimal delivery methods for this therapeutic agent. Dissolution studies revealed that the inclusion of cations facilitated the release of the drug. The duration and kinetics of drug release were also dependent on the characteristics of the polymers, cations, their concentration, and valence. Notably, the combination of the two polymers resulted in a more significant reduction in the drug release rate compared to formulations containing each polymer separately.

**Figure 8:**
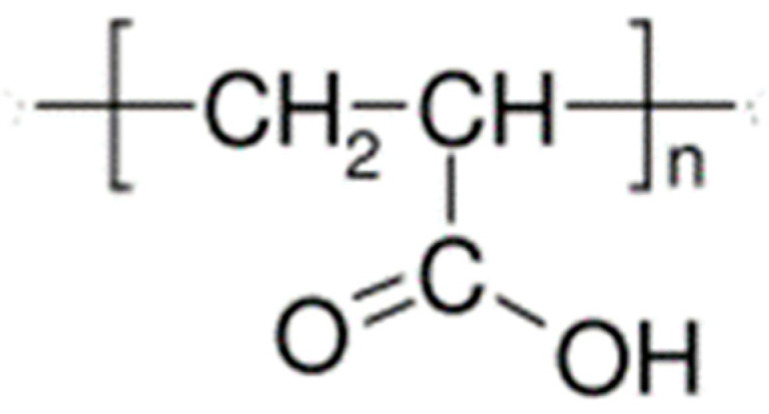
Polyacrylic acid.

Furthermore, Boaz Mizrahi [59], building upon previous research [85,86,87], focused on the development of a mucoadhesive tablet using the commonly used pharmaceutical polymers hydroxypropyl cellulose and crosslinked polyacrylic acid (PAA) (Figure 8). The tablet was designed to release citrus oil and magnesium chloride.

The tablet exhibited remarkable efficacy in reducing pain and promoting faster healing in patients with both single ulcers and recurrent aphthous stomatitis. The polymer capsule’s erosion time and flexibility demonstrated its ability to alleviate pain by shielding the ulcerative wound from the oral environment. In addition to the discomfort and potential nausea associated with many antimicrobial mouthwash solutions, the author and a colleague [60] explored iodine complexes with hydroxypropyl cellulose (HPC) (Figure 7) and ethyl cellulose (EC) as carriers for iodine in buccal dosage forms. The research involved investigating the release profile of iodine from adhesive tablets and assessing their antimicrobial activity through diffusion assays using Candida albicans and Porphyromonas gingivalis cultures [88]. The study revealed the unique binding and release properties of these polymers in relation to iodine, with EC demonstrating superior complexation capabilities compared to HPC.

**Figure 9:**
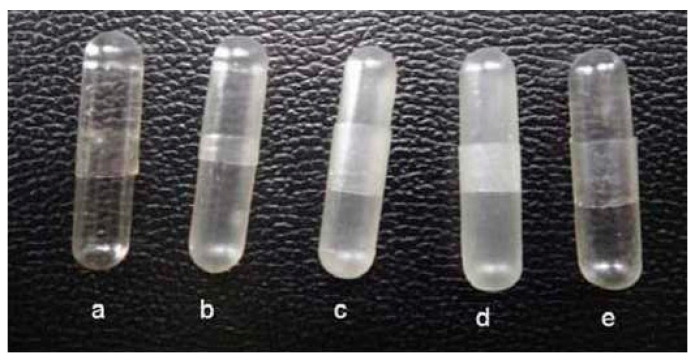
Photographs of capsules made from the solutions with different HPMC/HPS contents [89]. 485 (**a**) 100:0; (**b**) 70:30; (**c**) 50:50; (**d**) 30:70; (**e**) 0:100.

The investigation [89] delves into the integration of polyethylene glycol (PEG) as both a plasticizer and a compatibilizer in the blends. The researchers conducted a thorough examination and development of equilibrated solutions with an elevated solids concentration (20%) to facilitate the production of hard capsules through the well-established dipping–drying technique, employing stainless steel mold pins (Figure 9). Various aspects of the HPMC/HPS blends, encompassing viscosity, transparency, tensile strength, water contact angle, SEM, and FTIR, underwent meticulous characterization. The findings revealed that the blend system exhibits immiscibility but demonstrates a discernible level of compatibility, particularly with the inclusion of PEG. The presence of hydroxypropylene groups on both cellulose and starch contributed to an improved compatibility between HPMC and the modified starch. It was observed that the contact angle of the films increased with higher HPS content, indicating a heightened hydrophilicity of starch HPS in comparison to HPMC. Additionally, the study identified intermolecular hydrogen bonding through the migration and overlapping of FTIR peaks.

**Figure 10:**
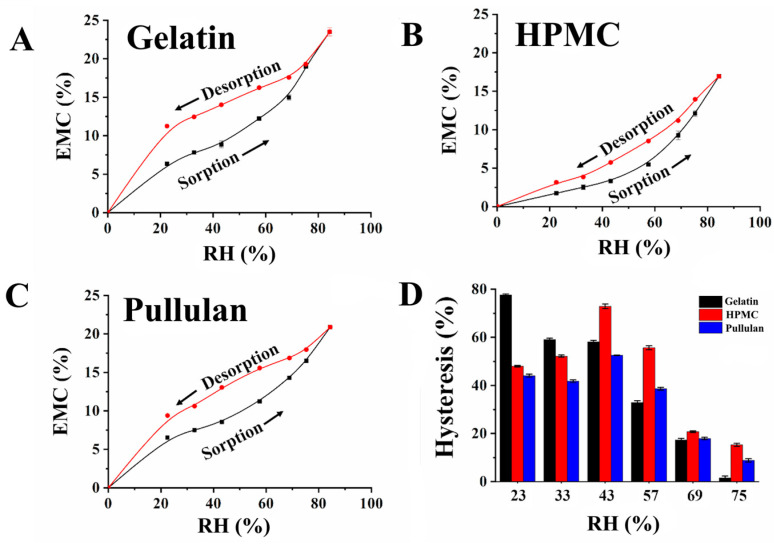
Moisture sorption and desorption circles for (**A**) gelatin, (**B**) HPMC, and pullulan capsules (**C**), and (**D**) the hysteresis values [91].

The study [90] examines the moisture sorption and desorption characteristics of three types of hard capsules: gelatin, HPMC, and pullulan capsules (Figure 10). The investigation delves into various aspects, including hygroscopicity, crystallinity, thermal behaviors, and related factors. The results indicate that HPMC capsules demonstrate lower moisture sorption rates, equilibrium moisture contents, moisture retention rates, and higher critical relative humidity compared to pullulan or gelatin capsules. Additionally, pullulan capsules exhibit a weaker moisture sorption ability but a comparable moisture retention capacity when compared to gelatin capsules. Notably, both HPMC and pullulan capsules demonstrate superior efficiency in safeguarding high, moderate, and low hygroscopic capsule contents (such as chitosan, potato starch, or ethyl cellulose) from external moisture absorption. Based on these findings, the study suggests that HPMC and pullulan capsules hold promise as viable alternatives to gelatin capsules derived from animal sources, primarily due to their favorable moisture sorption and desorption properties. 

In their article [52], the authors conducted a study utilizing EC/(PAA) and employed dynamic light scattering and transmission electron microscopy techniques. By selecting an appropriate solvent, the authors successfully achieved intra- and intermolecular association of the main chain in densely grafted copolymers, leading to the formation of micelles. The conformation of the chain in solvents served as an illustrative example of the influence of the solvent and graft length. This finding offers a strategy for controlling the micellization of grafted copolymers by adjusting the chain conformation.

In another study [91], the complexation between PAA and methylcellulose (MC) (Figure 7) in aqueous solutions was analyzed using the layer-by-layer deposition method of polymers on glass surfaces with Biacore, a system designed for the analysis of biological interactions.

It is noteworthy that Biacore analysis has become a valuable tool for quantifying drug–protein binding, characterizing antibodies, assessing immunogenicity, and developing vaccines, which are highly relevant research topics in today’s scientific landscape. The results obtained in the aforementioned article subsequently facilitated the optimization of experimental conditions for the sequential layering of films on slides.

In another study [75], the researchers investigated films prepared by pouring polymer solutions mixed with a drug at a pH of 4.5. The in vitro release of riboflavin from these films was studied using rabbit models. It was observed that films with a higher content of MC exhibited significantly slower release of riboflavin compared to samples containing higher amounts of PAA. Films composed of PAA/MC interpolymer complexes (IPCs) demonstrated relatively good adhesion and retention for 30–60 min, whereas films made solely of methylcellulose lasted for 50 min, and those made of PAA lasted only 10 min.

Chitosan has been extensively studied in various research endeavors. For instance, in the scientific article by N.A. Nafi et al. [69], mucoadhesive patches were prepared using polyvinyl alcohol (PVA), HEC, and chitosan as carriers for the delivery of cetylpyridinium chloride.

Additionally, Athos Maleki et al. [92,93] conducted research providing new insights into the impact of stable shear flows on intramolecular and intermolecular associations in dilute aqueous solutions of HEC in the presence of a crosslinking agent. The results highlighted that by adjusting the shear rate, crosslinking density, and polymer concentration, both intrapolymer and interpolymer crosslinking effects could be observed. A significant conclusion from these studies was that the hydrophobicity and concentration of the polymer can be manipulated as variables to regulate the compression of the gel matrix in the post-gel region. This effect can be employed to modulate the porosity of the hydrogel and consequently control the release characteristics of drugs in controlled drug delivery systems.

Mun G.A. and his colleagues [94,95,96,97] used viscometry and turbidimetry to study the formation of complexes between PAA/HEC, PAA/HPC, PAA, and poly(2-hydroxyethylacrylate) in media with different pH values. As a result of experiments, it was revealed that, depending on the critical pH of the solution, both hydrophilic associates and hydrophobic interpolymer complexes can form in these systems. Comparing the results of the turbidimetry method, we can say that the PAA/HPC system prevails with a great ability for complex formation compared to the PAA/HEC system. When inorganic salts are added to solutions of PAA/HEC polymers in acidic media, a polycomplex with a compact structural organization is formed, and the resulting films were considered as capsules for controlled isolation of the antibiotic levomecitin.

In the article [50], a more comprehensive investigation was conducted on hydrogels derived from PAA and HPC. The composition of the hydrogels, including the percentages of the initial polymers and crosslinking agents, was varied, and the swelling capacity of the hydrogels was evaluated. Hydrogels with higher swelling percentages were further characterized using techniques, such as IR spectroscopy, differential scanning calorimetry, dynamic mechanical analysis, and scanning electron microscopy. The most favorable swelling results were obtained with the PAA/HPC 50/50 sample, which was attributed to the interaction between divinyl sulfone (DVS) and the PAA chain as the preferred crosslinking agent for HPC in this system, contrary to the initial assumption of using glutaraldehyde. This gel, being partially biodegradable due to the presence of HPC, exhibited viscoelastic properties similar to PAA.

In the articles [72,73,98], the study focused on PAA/HPC hydrogels loaded with drugs, such as oxaliplatin, known for its potent antitumor activity, and bovine serum albumin, serving as an antigen for assessing changes in immune response. The release profiles indicated that the polymer composition played a crucial role in the drug release behavior. In vitro cytotoxicity analysis [99] yielded positive results. Moreover, based on these polymers, vesicles were also developed [100]. Vesicles are essential structures used as models for simulating living cells and as drug carriers, featuring a spheroidal two-layer shell. The dynamic formation process, encompassing the nucleation and growth of PAA/HPC bubbles, was observed, which differed from theoretical predictions regarding closed membrane models.

The article [101] presents a method for creating films by blending HEC and maleic acid ester of methyl vinyl ether. The resulting films exhibit favorable mechanical and physical characteristics when subjected to relatively mild heat treatment. The heat treatment induces crosslinking through intermolecular esterification and anhydride formation. The crosslinked materials demonstrate water-swelling capabilities, and the extent of swelling can be easily controlled by adjusting the temperature and duration of the heat treatment. This control over swelling is crucial for film modification.

In the research conducted by Kwaben Ofori-Kwakye and John Fell [102], mixed films containing pectin, chitosan, and HPMC were investigated with regard to polymer leaching in gastrointestinal fluids and its impact on the integrity of film-coated products during passage through the gastrointestinal tract. The ability to control the leaching of pectin in different pH conditions makes film coating systems potential carriers for two-phase drug delivery. Three years later, in [103], the authors explored the systematic complexation between polycarboxylic acids and HPMC. The study revealed that HPMC exhibits a comparable complexing ability to PAA, a lower ability compared to MC, higher ability than HPC, and a higher ability than HEC. In [104,105], the phase transition behavior of the HPMC/PAA system in water was investigated, attributing the decrease in transition temperature to strong hydrogen bonding between these polymers. At an acidic pH, interpolymer complexes were formed through hydrogen bonding at a specific stoichiometric ratio (3:1) of polymers. With an increase in pH, a transition to the formation of interpolymer associates was observed, accompanied by gelation.

Moreover, in the works [49,106,107] interpolymer complexes in dilute aqueous solutions were obtained and studied using viscometry, turbidimetry, and potentiometry, focusing on HPC and a copolymer of maleic acid and styrene. The investigations revealed the formation of stable interpolymer complexes due to strong physical interactions between the functional groups of these polymers. The total polymer concentration in the polycomplex solutions influenced the release profile of procaine, particularly when one of the components was present in excess.

In the articles [51,108], experimental data concerning complexation between HPC and polyacrylonitrile were presented. Various analytical techniques including viscometry, polarized optical microscopy, scanning electron microscopy, infrared spectrophotometric analysis, and thermogravimetric analysis were employed in this study. The results demonstrated excellent compatibility within the mixture system, likely attributable to strong hydrogen bonding interactions between the hydroxyl group of HPC and the nitrile group of polyacrylonitrile. The interaction parameters, such as Huggins’ constants and association constants, calculated based on viscosity measurements, provided reliable information, and proved to be valuable tools for characterizing the compatibility of polymer mixtures.

In the research conducted by Rubner et al. [109], the formation of multilayers composed of two weak polyelectrolytes, PAA and polyallylamine, was investigated under different pH conditions. Similarly, Bjorn Scheler, Evgeny Poptoshev, and Frank Caruso [110] examined the structure of these multilayer films comprising a low but consistently charged polyelectrolyte and the weak polyacid PAA, considering cases where PAA had a low or high charge. The charge density of PAA was modified by adjusting the pH of the deposition solutions, demonstrating that the magnitude of electrostatic and secondary interactions in multilayer polyelectrolyte films can be controlled by varying the charge density of the polyelectrolyte in response to the solution’s pH.

In the research conducted by Yuwei Zhang et al. [111,112,113,114], non-covalently linked micelles with a polycaprolactone core and a shell based on PAA were obtained in aqueous solutions due to specific interactions between the constituent polymers. Additionally, thin films composed of water-soluble cellulose derivatives were developed. The study highlighted the potential utility of cross-linked micelle shells and hollow spheres for encapsulation in various fields, particularly in biomedicine. The films exhibited excellent water absorption properties and pH-sensitive behavior, making them suitable for applications in biomedicine and oil purification from water in the oil industry.

Nanoparticles incorporating HPC with thermal sensitivity and carboxylic functional groups were investigated at Shanghai University in the absence of surfactants and organic solvents [115,116,117], and with the application of surfactants and organic solvents [118]. In the first system, rapid and reversible changes in dispersion–aggregation behavior were observed, closely related to temperature variations within a narrow range near body temperature. This phenomenon was confirmed through dynamic light scattering analysis. In the second system, the size and morphology of the nanoparticles formed could be manipulated by adjusting the ratio of HPC and the films. The authors of these studies concluded that utilizing HPC as a renewable central material within a favorable solvent medium offers numerous advantages, ranging from environmental safety to their applicability in biologically significant systems.

In the article [63,119], the pH-dependent micellization and crosslinking of HEC/PAA nanoparticles were comprehensively investigated. The loading of crosslinked nanoparticles with drugs was also explored. Dynamic light scattering studies revealed that the hydrodynamic diameter of the pH-induced micelles was influenced by temperature and concentration. Additionally, the potential of crosslinked micelles for loading cationic drugs was demonstrated using diminazene as a model drug.

In [70], novel drug delivery systems based on HPC and varying percentages of glucose were developed and characterized to assess their suitability as patches. The primary objective of the study was to investigate and minimize changes that may occur when HPC is used as a patch or bandage on skin exposed to ultraviolet (UV) light. The total amount of glucose released from the HPC matrix was primarily influenced by the amount of glucose incorporated and, to a lesser extent, the duration of UV radiation exposure. Morphological and surface energy analyses indicated the occurrence of photooxidation and photodegradation processes, leading to chemical modifications on the polymer surface.

In the article [120], a novel drug carrier for the colon based on a natural polymer was developed using carboxymethylcellulose and acrylic acid in an aqueous solution. The impact of various synthesis parameters, including the content of natural polymer and radiation dose, on the gelation process was investigated. The swelling behavior of the prepared hydrogels was characterized by studying the time- and pH-dependent swelling of hydrogels with different carboxymethylcellulose content. The swelling kinetics studies revealed that the hydrogel exhibited Fickian diffusion in an environment similar to the stomach (pH 1) and also in an environment resembling the intestine (pH 7).

Researchers from Kyoto University conducted a study [121] focusing on cellulose and its derivatives. In this work, polycomplexes were obtained by casting from mixed polymer solutions. It was observed that complex formation was driven by the increased frequency of hydrogen bonding interactions between the residual hydroxyl groups of the initial polymers. Considering the wide applications of cellulose derivatives in industries, such as petroleum, textiles, food, pharmaceuticals, and others, further research in this area remains relevant.

Solid dispersion, which involves dispersing compounds in water-soluble carriers, is commonly used to enhance the dissolution properties and bioavailability of poorly water-soluble drugs. Based on this concept, researchers from Tokyo University of Pharmacy and Life Sciences conducted a study [64] on the controlled release of phenacetin from solid dispersion by forming an interpolymer complex between methylcellulose and carboxyvinyl polymer. The research demonstrated the feasibility of controlling the release of phenacetin from solid dispersion granules by modulating the formation of a complex between the starting polymers, which can be achieved by adjusting the ratio and molecular weight.

Considering the significant advancements highlighted in multiple works [122,123,124,125], the production of interpolymer complexes stabilized by hydrogen bonding through the layer-by-layer deposition method is a highly relevant area of study. In a scientific investigation [126], the authors focused on the study of multilayer film coatings obtained by depositing polyacrylic acid-based polymers with nonionic polymers onto glass surfaces. Chemical modification of the glass surface, followed by layer-by-layer deposition and crosslinking of interpolymer complexes through heat treatment, enabled the production of ultra-thin film coatings that remain adhered to the substrate. The thickness of these coatings is directly influenced by the number of deposition layers and crosslinking conditions. An intriguing dependence of the swelling properties on the thickness of the film was observed, possibly attributed to gradual transitions between the layers. Such coatings have potential applications as substrates for investigating the adhesive properties of pharmaceutical tablets and simulating the overall adhesion observed during the detachment of mucoadhesives from mucous membranes, among other uses.

The process of adsorbing a polymer solution onto a solid surface is widely employed for film production, yet the forces governing adsorption are not fully understood. In the article [127], the authors investigated the adsorption of cellulose surfactants on polymethylmethacrylate (PMMA) surfaces. This study provides a comprehensive understanding of how surface-active molecules interact with solid surfaces and self-organize into distinct architectures. The research revealed that regardless of the complex interactions between the adsorbed molecules and the solid surface, hydrogen bonding plays a fundamental role in the spontaneous adsorption on PMMA surfaces. Hydrogen bonding leads to strong attachment of the molecules to the surface, resulting in irreversible adsorption that cannot be removed by water washing. In contrast, in the absence of hydrogen bonding, the molecules weakly adhere to the surface, leading to reversible adsorption. These findings enable predictable manipulation of adsorption and self-assembled architectures by tailoring the molecular structures and solid surfaces.

In the layer-by-layer deposition method, surface functionality plays a crucial role in the deposition of polymer complexes. Hydrophobic surfaces in contact with water do not provide accurate predictions of the surface’s ability to be covered with water-soluble polymer complexes. The chemical composition of the surface also influences the deposition efficiency, and surfaces that are prone to hydrogen bonding facilitate the deposition of hydrogen-bonded polymer complexes. Importantly, surface properties are not solely affected by the initial polymer deposition layer but can also propagate and impact the properties of the resulting multilayer film on the surface.

Molecular dynamics modeling was employed in the study described in [128] to investigate the aggregates formed by different polymers in aqueous solutions. The investigation focused on hydrolyzed polyacrylamide (PAM), HEC, and polyvinylpyrrolidone (PVP). The structures of mixed aggregates were analyzed based on dihedral angular distribution, comparing PAM in pure form, PAM in aqueous solution, and various ratios with HEC and PVP. The research confirmed the presence of strong interaction between PAM and HEC and provided additional microscopic insights into these systems through spectral analyses.

The article [129] describes the production of a volumetric polymer complex bound by hydrogen bonds and thin films through solution mixing and layer-by-layer assembly. PVP and ethylene polyoxide served as hydrogen bond acceptor polymers, while PAA and PMMA acted as hydrogen bond donor polymers. When immersed in solvent systems, the PVP/PAA films showed that as hydrogen bonds break, the polymer chains easily detach from the film, leading to film thinning. Comparatively, the dissolution of bulk polymer complexes became more challenging due to the need to break hydrogen bonds and overcome polymer chain entanglement.

Films formed from ethylene polyoxide and PAA were also explored for three-dimensional (3D) integrated optics using customizable multi-level building blocks, demonstrating their usefulness in manipulating polarized light across a broad spectrum [53].

## 6. Conclusions

In conclusion, extensive research has been conducted on various polymer combinations as highly specific functional materials, leading to the successful synthesis of IPCs from different starting materials using diverse methods, both in solution and on different surfaces. Experimental investigations have revealed that the formation of IPCs is influenced by factors, such as pH, temperature, nature, and the ratio of initial polymers. To achieve effective functionalization and growth of polycomplexes in solution and surface-based processes, it is important to consider the presence of impurities and monomer particles from the initial polymers. These findings suggest that further modifications in IPC synthesis methods have the potential to enhance performance in industrial processes and expand the range of applications for IPCs.

Furthermore, cellulose ethers, including HEMC and HPMC, have gained significant use in the manufacturing of contemporary dry building mixes and pharmaceutical dosage forms. These polymers offer advantages, such as improved water retention, thickening properties, and enhanced processability of mixtures. Moreover, they exhibit the capability to prolong the residence time of dosage forms on mucous membranes, leading to increased bioavailability of medications. The development of IPCs using cellulose derivatives and other polymers has attracted attention due to their biocompatibility and potential for biomedical applications. IPCs demonstrate a wide range of properties, spanning from glassy to elastic rubber-like materials, making them suitable for diverse applications, such as ophthalmology and local drug delivery. The utilization of layer-by-layer deposition techniques has facilitated the fabrication of tailored multilayer films, further expanding the potential of IPCs in drug delivery systems.

Additionally, research has focused on exploring the mucoadhesive properties of cellulose-based formulations, showcasing their efficacy in treating conditions, such as ulcerative wounds, acne rosacea, and periodontal diseases. Investigations into the incorporation of polyvalent cations and the optimization of polymer combinations have been conducted to regulate drug release rates and enhance antimicrobial activity. Overall, these studies contribute to the understanding and advancement of cellulose-based materials for biomedical applications, offering promising avenues for future research and innovations in the field of drug delivery systems.

## Figures and Tables

**Table 1:** Cellulose and its derivatives (Figure 6).

Cellulose Esters	R Groups	Cellulose Ethers	R Groups
Acetate	H, I	Methylcellulose	H, CH_3_
Acetate trimellitate	H, I, II	Ethyl cellulose	H, CH_2_CH_3_
Acetate phthalate	I, III	Hydroxyethylmethyl cellulose	H, CH_3_, [CH_2_CH_2_O]nH
Hydroxypropyl methyl phthalate	H, CH_3_, CH_2_CH(OH)CH_3_, III, IV	Hydroxypropyl cellulose	H, [CH_2_CH(CH_3_)O]nH
Hydroxypropyl methyl phthalate acetate succinate	H, CH_3_, CH_2_CH(OH)CH_3_, I, V	Carboxymethylcellulose	H, CH_2_COONa

**Table 2:** Methods of delivery of various medicines.

Dosage Forms	Drugs and Medicinal Products	Links
Mucoadhesive tablets	Metronidazole; chlorhexidine diacetate; theophylline; magnesium chloride; diminazene; phenacetin; iodine.	[56,57,58,59,60,61,62,63,64,65]
Medical patches	Antimicrobial biomaterials; 5-fluorouracil; cetylpyridinium chloride.	[66,67,68,69,70]
Hydrogels	Metronidazole; oxaliplatin, Voltaren Emulgel (carbomer and propylene glycol alginate)	[71,72,73]
Eye drops	Riboflavin	[74,75]
Topical creams and ointments:	Diclofenac sodium topical gel	[76]
Inhalation products	Beclomethasone dipropionate inhaler	[77]

## Data Availability

Not applicable.
